# Rare Extrahepatic Metastasis of Hepatocellular Carcinoma to the Left Triangular Ligament: A Case Report

**DOI:** 10.70352/scrj.cr.26-0217

**Published:** 2026-06-03

**Authors:** Ayumi Tsuda, Takehiko Hanaki, Hiroshi Ishii, Kentaro Uehira, Jun Yoshida, Mikiya Kishino, Masaki Morimoto, Yuki Murakami, Naruo Tokuyasu, Teruhisa Sakamoto, Yoshiyuki Fujiwara

**Affiliations:** 1 Division of Gastrointestinal and Pediatric Surgery, Tottori University Faculty of Medicine, Yonago, Tottori, Japan; 2 Department of Surgery, Tottori Prefectural Central Hospital, Tottori, Tottori, Japan; 3 Division of Medical Education, Tottori University Faculty of Medicine, Yonago, Tottori, Japan; 4 Department of Pathology, Tottori University Faculty of Medicine, Yonago, Tottori, Japan

**Keywords:** hepatocellular carcinoma, extrahepatic metastasis, left triangular ligament, triangular ligament metastasis, laparoscopic hepatectomy

## Abstract

**INTRODUCTION:**

The left triangular ligament is an uncommon site of heterotopic liver tissue, from which ectopic hepatocellular carcinoma (HCC) may arise. In contrast, extrahepatic metastasis from an intrahepatic primary HCC to the triangular ligament appears to be extremely rare. To the best of our knowledge, within the scope of our structured search of the English-language literature (PubMed and Web of Science), we did not identify a pathologically confirmed report of an isolated metastasis to the left triangular ligament from an orthotopic HCC. We report a case of isolated HCC metastasis to the left triangular ligament discovered incidentally during laparoscopic hepatectomy, which posed a differential diagnostic challenge between ectopic HCC and extrahepatic metastasis.

**CASE PRESENTATION:**

A 62-year-old man was referred for evaluation of increasing serum alpha-fetoprotein (AFP) levels (27772 ng/mL). Dynamic contrast-enhanced CT revealed an approximately 25-mm hypervascular tumor in segment 2 with washout, consistent with HCC, and laparoscopic left lateral sectionectomy was planned. Intraoperatively, during mobilization of the left lateral section, an unexpected flat, white lesion was identified within the left triangular ligament, adjacent to but clearly separate from the primary tumor and liver parenchyma. Because malignancy could not be excluded, the triangular ligament was transected on the diaphragmatic side and resected en bloc with the planned specimen. Histopathological examination showed the segment 2 tumor to be well- to moderately differentiated HCC, whereas the triangular ligament lesion consisted of poorly differentiated HCC. The lesion was located entirely within the peritoneal fold, enclosed by peritoneal tissue, without intervening normal hepatic parenchyma or macroscopic/microscopic continuity with the primary tumor. These findings supported the diagnosis of isolated extrahepatic metastasis rather than ectopic HCC. The final pathological stage was pT2N0M1, pStage IVB (Union for International Cancer Control TNM, 8th edition). The postoperative course was uneventful, and the patient has remained recurrence-free with normalized AFP for 5 years.

**CONCLUSIONS:**

This case demonstrates an unusual pattern of HCC spread to the left triangular ligament, a site also known for ectopic HCC. Accurate diagnosis required integration of intraoperative findings and histopathological features. Careful inspection of the triangular ligament during left-sided mobilization should be considered to detect occult lesions and facilitate complete resection.

## Abbreviations

AFP
: alpha-fetoprotein
HCC
: hepatocellular carcinoma

## INTRODUCTION

The left triangular ligament is a recognized but uncommon site of ectopic HCC, where heterotopic liver tissue may give rise to a primary tumor in the absence of an orthotopic hepatic lesion.^1)^ In contrast, extrahepatic metastasis from intrahepatic HCC to this peritoneal fold appears to be extremely rare.^2,3)^

HCC most commonly recurs within the remnant liver after curative treatment. In contrast, extrahepatic metastases are less frequent, with a cumulative incidence of approximately 13% at 5 years during the clinical course after treatment, and typically involve the lungs, lymph nodes, bone, or adrenal glands.^4–6)^ Metastatic spread to the perihepatic peritoneal folds is seldom reported and is usually associated with widespread peritoneal dissemination or direct diaphragmatic invasion.^7,8)^

Herein, we report a rare case of HCC in segment 2 (S2) in which an unexpected separate lesion was incidentally identified within the left triangular ligament during laparoscopic left lateral sectionectomy. Histopathological examination ultimately confirmed the lesion as an isolated extrahepatic metastasis from the orthotopic primary tumor, posing an important differential diagnostic challenge compared with ectopic HCC arising in the same location.

## CASE PRESENTATION

A 62-year-old man had been followed at a local clinic for 2 years for obesity and fatty liver disease. During routine follow-up, a progressive increase in serum AFP was noted, and he was referred to the Tottori University Hospital for further evaluation. He was asymptomatic at presentation.

**Fig. 1:**
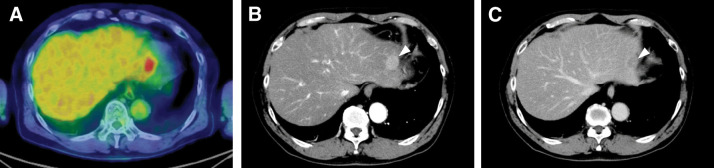
Preoperative PET-CT and dynamic CT findings of the primary hepatic lesion. (**A**) PET-CT demonstrated FDG uptake in the primary lesion (SUVmax 5.03). No definite abnormal uptake corresponding to the triangular ligament lesion was identified preoperatively. (**B**) Dynamic contrast-enhanced CT demonstrated an approximately 2.5-cm tumor at the peripheral portion of the left lateral section, showing arterial-phase hyperenhancement (arrowhead). (**C**) On the delayed/equilibrium phase, the same lesion showed washout (arrowhead), consistent with hepatocellular carcinoma. FDG, fluorodeoxyglucose; SUVmax, maximum standardized uptake value

**Fig. 2:**
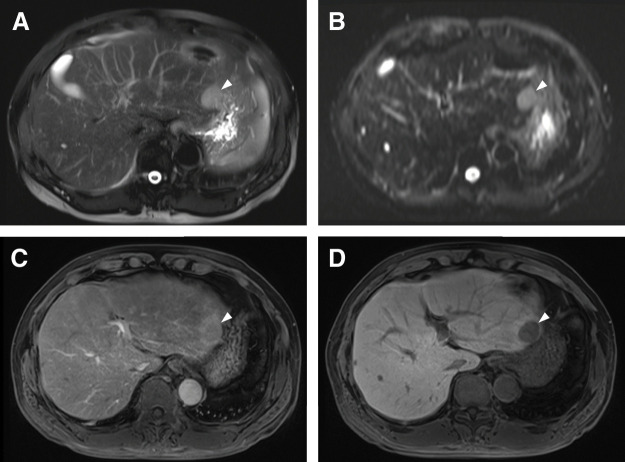
Preoperative MRI findings of the primary hepatic lesion. (**A**) T2-weighted fat-suppressed HASTE image showed a 3.0-cm hyperintense lesion at the periphery of the left lateral section (arrowhead). (**B**) Diffusion-weighted image (b = 800 s/mm^2^) showed the lesion as hyperintense (arrowhead). (**C**) Arterial-phase dynamic contrast-enhanced MRI showed mild hyperenhancement of the lesion (arrowhead). (**D**) In the hepatobiliary phase obtained 20 min after contrast administration, the lesion appeared hypointense relative to the surrounding liver parenchyma (arrowhead). HASTE, half-Fourier acquisition single-shot turbo spin echo

PET-CT showed fluorodeoxyglucose uptake in the left lateral section (**Fig. 1A**). Dynamic contrast-enhanced CT demonstrated a 25-mm tumor in S2 with arterial-phase hyperenhancement and equilibrium-phase washout, radiologically consistent with HCC (**Fig. 1B** and **1C**). Preoperative MRI demonstrated a 3.0-cm lesion at the periphery of the left lateral section. The lesion was hyperintense on T2-weighted fat-suppressed half-Fourier acquisition single-shot turbo spin echo imaging and diffusion-weighted imaging, showed mild hyperenhancement in the arterial phase of dynamic contrast-enhanced MRI, and appeared hypointense relative to the surrounding liver parenchyma in the hepatobiliary phase obtained 20 min after contrast administration (**Fig. 2A**–**2D**). No additional lesion other than the primary hepatic lesion was identified on preoperative imaging, including contrast-enhanced CT, PET-CT, and MRI.

He was referred to the Division of Gastrointestinal and Pediatric Surgery at Tottori University Hospital for surgical resection with a preoperative diagnosis of solitary HCC (cT1N0M0, cStage I, Union for International Cancer Control [UICC] TNM classification, 8th edition).

His medical history included dyslipidemia, type 2 diabetes mellitus, and ureteral stones. He consumed alcohol occasionally and had a 41 pack-year smoking history. There was no family history of liver disease, although his grandmother had gastric cancer.

Physical examination revealed an obese abdomen (BMI 27.4 kg/m^2^) without tenderness or hepatomegaly. Laboratory findings showed preserved liver function (Child–Pugh class A, indocyanine green retention rate at 15 minutes 20%). AFP was markedly elevated at 27772 ng/mL (institutional upper limit of normal: <10 ng/mL), while carcinoembryonic antigen and carbohydrate antigen 19-9 were normal. Serologic testing showed hepatitis B surface antigen negativity with positivity for anti-hepatitis B surface and anti-hepatitis B core, and anti-hepatitis C virus antibody was negative.

**Fig. 3:**
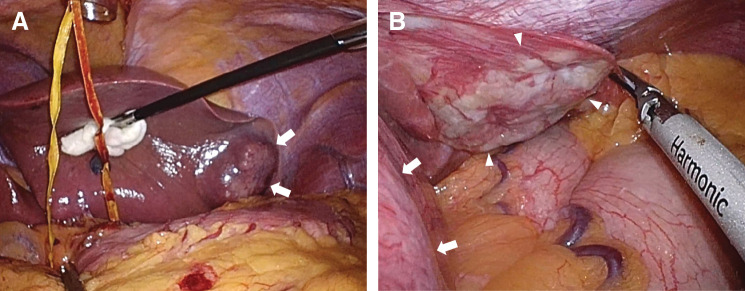
Intraoperative findings during laparoscopic left lateral sectionectomy. (**A**) Intraoperative view from the inferior aspect of the left lateral section showing the primary tumor in the left lateral section that had been identified preoperatively (arrows). (**B**) A flat, white lesion (arrowheads) was identified within the left triangular ligament, which was located lateral to the primary tumor (arrows).

**Fig. 4:**
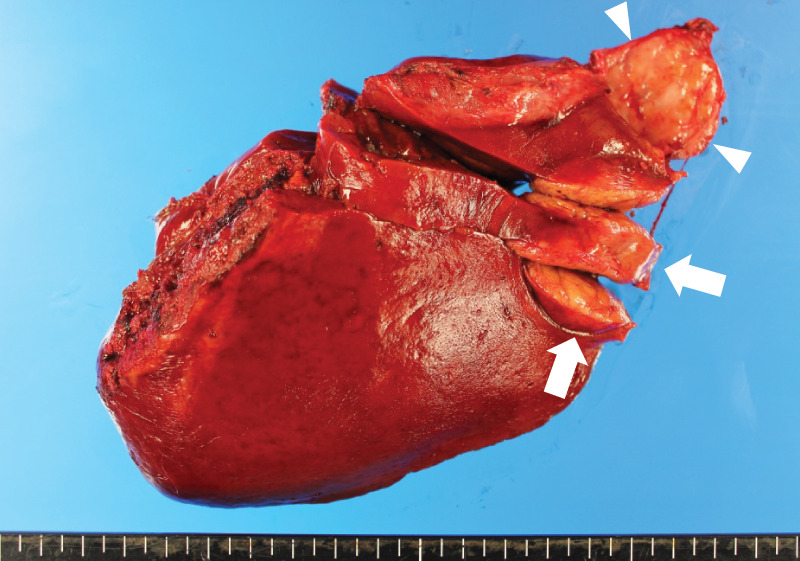
Macroscopic findings of the resected specimen. Arrows indicate the primary tumor (approximately 4.5 cm in greatest dimension). Arrowheads indicate the flat lesion in the left triangular ligament. Note that the specimen had already been sectioned before photography.

Laparoscopic left lateral sectionectomy was planned. Intraoperatively, the S2 tumor was identified as expected (**Fig. 3A**). However, during mobilization of the left lateral section, an unexpected flat, white lesion was incidentally discovered within the left triangular ligament adjacent to the primary tumor but clearly separate from it (**Fig. 3B**). Because preoperative imaging had not clearly delineated this lesion as a distinct entity and malignancy could not be excluded, the left triangular ligament was transected on the diaphragmatic side to secure an adequate margin, and the lesion was resected simultaneously with the planned left lateral sectionectomy (**Fig. 4**). Operative time was 2 h 26 min with 50 mL blood loss.

The postoperative course was uneventful, and the patient was discharged on POD 7.

**Fig. 5:**
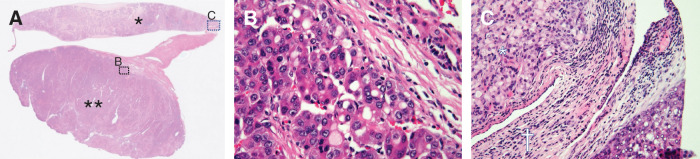
Histopathological findings (hematoxylin and eosin staining). (**A**) Low-power overview of the resected specimen. * indicates the flat lesion in the left triangular ligament, and ** indicates the primary intrahepatic tumor. Dotted boxes indicate the areas shown in (**B**) and (**C**). (**B**) Higher-magnification view of the primary tumor, showing well- to moderately differentiated HCC. (**C**) Higher-magnification view of the triangular ligament lesion (*) and adjacent triangular ligament tissue (^†^). No non-neoplastic hepatic parenchyma was identified in the triangular ligament tissue (^†^), and the lesion (*) was entirely composed of tumor tissue without intervening normal liver tissue. Microscopic examination of all examined sections confirmed that the lesion was confined to the triangular ligament and showed no continuity with the adjacent liver parenchyma. The lesion was enclosed by peritoneal tissue within the triangular ligament, favoring an extrahepatic (intraligamentous) metastatic lesion rather than ectopic HCC. HCC, hepatocellular carcinoma

**Fig. 6:**
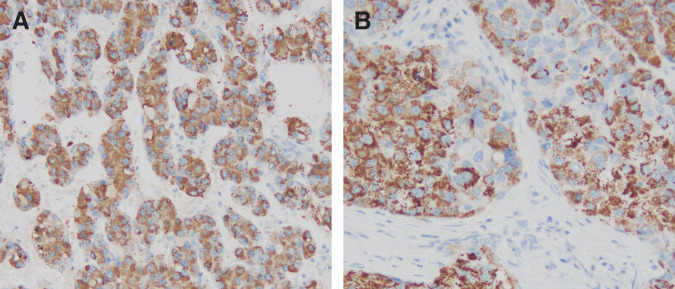
Immunohistochemical findings (HepPar-1 staining). (**A**) Higher-magnification view of the primary tumor, showing positive HepPar-1 immunoreactivity. (**B**) Higher-magnification view of the triangular ligament lesion, also showing positive HepPar-1 immunoreactivity. These findings supported hepatocellular differentiation in both lesions. HepPar-1, hepatocyte paraffin 1

Histopathological examination revealed that the S2 tumor (45 × 30 × 45 mm) was a well- to moderately differentiated HCC with trabecular and solid growth, focal pseudoglandular formation, a fibrous capsule, and vascular invasion (vp1, vv1, va1). The border between the primary tumor and the surrounding normal liver parenchyma was clearly identifiable histologically. The triangular ligament lesion consisted of poorly differentiated HCC without intervening normal hepatic parenchyma. Microscopic examination of all examined sections confirmed that the lesion was completely confined to the left triangular ligament and showed no continuity with the adjacent liver parenchyma. It was enclosed by peritoneal tissue and showed no macroscopic or microscopic continuity with the primary tumor (**Fig. 5A**–**5C**). Additional immunohistochemical staining showed hepatocyte paraffin 1 positivity in both the primary lesion and the triangular ligament lesion (**Fig. 6A** and **6B**). Arginase-1 showed focal positivity in the primary lesion and positivity in the triangular ligament lesion. These findings supported hepatocellular differentiation in both lesions. The discrepancy between the CT-based and pathological tumor size may reflect differences in imaging modality and measurement plane. In addition, interval tumor growth between CT, MRI, and surgery cannot be completely excluded. The non-tumorous liver showed chronic hepatitis with mild fibrosis (CH, f1). These histopathological findings favored an isolated extrahepatic (intraligamentous) metastasis rather than an intrahepatic lesion or direct extension. Based on these findings, the triangular ligament lesion was diagnosed as an isolated extrahepatic metastasis from HCC. The final pathological diagnosis was pT2N0M1, pStage IVB (UICC TNM). Although the lesion was classified as M1 under the UICC TNM system because it was extrahepatic, it was intraligamentous, solitary, and completely resectable, which should be considered when interpreting the favorable long-term outcome.

The patient has remained recurrence-free with normal AFP levels for 5 years postoperatively.

## DISCUSSION

This case illustrates a rare presentation of HCC: an isolated lesion within the left triangular ligament identified during resection of an orthotopic tumor in S2. The left triangular ligament is an uncommon site for ectopic HCC arising from heterotopic liver tissue.^1,9,10)^ In contrast, extrahepatic metastasis from an intrahepatic primary HCC to this peritoneal fold appears to be exceedingly rare.^2,3)^ To the best of our knowledge, our structured search did not identify a pathologically confirmed report of isolated metastasis to the left triangular ligament from an orthotopic HCC (**Supplementary Material 1**).

The differential diagnosis between ectopic HCC and extrahepatic metastasis was central to this case. Key histopathological features favored metastatic disease: the lesion was located entirely within the peritoneal fold and was completely enclosed by peritoneal tissue without intervening normal hepatic parenchyma or macroscopic/microscopic continuity with the primary tumor. In addition, microscopic examination of all examined sections confirmed that the lesion showed no continuity with the adjacent liver parenchyma. Although the anatomical proximity of the left triangular ligament to the liver surface, as well as the presence of portal venous invasion (vp1) in the primary tumor, made this distinction particularly important, the histopathological distribution in this case was considered more consistent with an isolated extrahepatic (intraligamentous) metastasis than with an intrahepatic lesion or direct extension. Ectopic HCC, by definition, arises from heterotopic liver tissue and therefore typically contains islands of normal hepatocytes within the lesion; the complete absence of such normal hepatic parenchyma in the present case strongly favored an isolated metastatic deposit rather than a *de novo* ectopic primary tumor.^1,11)^ The triangular ligament lesion showed no definite fluorodeoxyglucose uptake on preoperative PET-CT. This is most plausibly explained by limited detectability due to partial-volume effects, given its small size and flat morphology; a biological contribution cannot be excluded but remains speculative. Although the immunohistochemical findings supported hepatocellular differentiation in both lesions, they did not by themselves determine the anatomical classification of the triangular ligament lesion. In the present case, the diagnosis of isolated extrahepatic metastasis was based primarily on the anatomical and histopathological distribution of the lesion. The mechanism of spread remains uncertain but may involve localized perihepatic dissemination, possibly through microscopic lymphatic or vascular routes along the ligament.

Surgically, the incidental intraoperative finding highlights the need for careful inspection during left lateral section mobilization. Preoperative imaging did not clearly identify the lesion as a separate focus, but transection on the diaphragmatic side allowed simultaneous complete resection with an adequate surgical margin.^12,13)^ This suggests that routine scrutiny of the triangular ligament and adjacent perihepatic structures may help detect occult disease in selected cases. Although this single case cannot establish a preventive effect, oncologically mindful handling of the triangular ligament may help reduce residual perihepatic disease near the diaphragm.

Despite pathological upstaging to pT2N0M1 (pStage IVB), the patient has remained recurrence-free. Although no general conclusion can be drawn from a single case, this course suggests the potential clinical relevance of complete resection of isolated extrahepatic foci when feasible.

Limitations include the single-case design, lack of molecular clonality analysis, and the possibility of unreported or non-English cases. Nonetheless, this report contributes to the understanding of rare extrahepatic spread patterns in HCC.

In summary, this case demonstrates an unusual pattern of HCC spread involving the left triangular ligament. It underscores the importance of histopathological evaluation in differentiating metastatic HCC from ectopic HCC at the same site and highlights the value of intraoperative vigilance during hepatectomy.

## CONCLUSIONS

This case describes an unusual and clinically important pattern of HCC spread: an isolated extrahepatic metastasis to the left triangular ligament identified during hepatectomy. Because the left triangular ligament is also a known site of ectopic HCC, accurate diagnosis requires careful integration of intraoperative and histopathological findings. Our experience suggests that meticulous inspection of the triangular ligament during left-sided liver mobilization may be important for detecting occult lesions and achieving complete resection in selected cases.

## SUPPLEMENTARY MATERIALS

Search history and search details via PubMed and Web of Science Purpose: To identify reports of HCC involving the left triangular ligament, with particular attention to metastatic lesions and distinguishing such cases from ectopic HCC arising in the same location.

## DECLARATIONS

### Availability of data and materials

All data generated or analyzed during this study are included within this published article.
