# Supplementary material for: Rare Extrahepatic Metastasis of Hepatocellular Carcinoma to the Left Triangular Ligament: A Case Report
Source: Surg Case Rep. 2026 Jun 3;12(1):26-0217. doi: 10.70352/scrj.cr.26-0217 (PMC13237782; doi:10.70352/scrj.cr.26-0217)
Supplement: Search history and search details via PubMed and Web of Science — Purpose: To identify reports of HCC involving the left triangular ligament, with particular attention to metastatic lesions and distinguishing such cases from ectopic HCC arising in the same location. [file scr-12-01-26-0217-s001.pdf]

Supplementary Material 1. Search history and search details via PubMed and Web of Science

Purpose: To identify reports of hepatocellular carcinoma (HCC) involving the left triangular ligament, with particular attention to metastatic lesions, and to distinguish such cases from ectopic HCC arising in the same location.

Search date: February 22, 2026
Database: **PubMed (NCBI)**

Search strategy and results
Search #1
Query: ("hepatocellular carcinoma"[Title/Abstract] OR HCC[Title/Abstract]) AND ("left triangular ligament"[Title/Abstract] OR "triangular ligament"[Title/Abstract]) AND (metastasis[Title/Abstract] OR metastatic[Title/Abstract] OR recurrence[Title/Abstract] OR spread[Title/Abstract])
Results: 1

Search #2
Query: ("hepatocellular carcinoma"[Title/Abstract] OR HCC[Title/Abstract]) AND ("left triangular ligament"[Title/Abstract] OR "triangular ligament"[Title/Abstract])
Results: 5

Search #3
Query: ("hepatocellular carcinoma"[Title/Abstract] OR HCC[Title/Abstract]) AND (ectopic[Title/Abstract] OR "ectopic liver"[Title/Abstract] OR "extrahepatic liver tissue"[Title/Abstract]) AND ("left triangular ligament"[Title/Abstract] OR "triangular ligament"[Title/Abstract])
Results: 1

Search #4
Query: ("Carcinoma, Hepatocellular"[Mesh] OR "hepatocellular carcinoma"[Title/Abstract] OR HCC[Title/Abstract]) AND ("triangular ligament"[Title/Abstract] OR "left triangular ligament"[Title/Abstract]) AND ("Neoplasm Metastasis"[Mesh] OR metastasis[Title/Abstract] OR metastatic[Title/Abstract])
Results: 0

Screening note (PubMed)
Titles and abstracts of retrieved records were reviewed to identify reports of:
- ectopic HCC arising in the left triangular ligament, and
- metastatic HCC to the left triangular ligament.

No well-documented case of isolated metastasis from intrahepatic HCC to the left triangular ligament was identified in the English-language literature through this PubMed search.

Search date: February 22, 2026
Database: **Web of Science Core Collection**
Search field: Topic (TS)

Search strategy and results

Search #1
Query: TS=(("hepatocellular carcinoma" OR HCC) AND ("left triangular ligament" OR "triangular ligament") AND (metastasis OR metastatic OR recurrence OR spread))
Results: 1

Search #2
Query: TS=(("hepatocellular carcinoma" OR HCC) AND ("left triangular ligament" OR "triangular ligament"))
Results: 4

Search #3
Query: TS=(("hepatocellular carcinoma" OR HCC) AND (ectopic OR "ectopic liver" OR "extrahepatic liver tissue") AND ("left triangular ligament" OR "triangular ligament"))
Results: 1

Search #4
Query: TS=(("hepatocellular carcinoma" OR HCC) AND ("left triangular ligament" OR "triangular ligament") AND (metastasis OR metastatic))
Results: 0

Search #5
Query: TS=(("hepatocellular carcinoma" OR HCC) AND ("left subdiaphragm" OR subdiaphragmatic OR diaphragmatic) AND (ectopic OR metastasis OR metastatic))
Results: 48

Screening note (Web of Science)
Records retrieved by Searches #1–#4 were screened to identify reports of:
- HCC lesions specifically involving the left triangular ligament,
- ectopic HCC arising in the left triangular ligament, and
- metastatic HCC to the left triangular ligament.
Search #5 was used as a broader supplementary search to identify:
- potentially related subdiaphragmatic/diaphragmatic HCC cases, and
- patterns of reported extrahepatic disease relevant to the differential interpretation of the present case.
No well-documented case of isolated metastasis from intrahepatic HCC to the left triangular ligament was identified in the English-language literature through this Web of Science search.
